# Facilitating Airway Surgery in a Morbidly Obese Patient Using Transnasal Humidified Rapid Insufflation Ventilatory Exchange (THRIVE)

**DOI:** 10.1155/2018/5310342

**Published:** 2018-11-19

**Authors:** Si-Jia Lee, Kelvin Howyow Quek

**Affiliations:** ^1^Department of Anaesthesia, Singapore General Hospital, Outram Road, Singapore 169608; ^2^Department of Anaesthesia, Changi General Hospital, 2 Simei Street 3, Singapore 529889

## Abstract

Transnasal Humidified Rapid Insufflation Ventilatory Exchange (THRIVE) is a relatively new noninvasive oxygenation technique with a broad range of applications. It is used in the treatment of type one respiratory failure, as a preoxygenation tool, as a rescue and temporising measure in difficult airways, and as step-down oxygen therapy in patients after extubation. Its use has also been described in laryngeal surgeries, but they mainly involved normal-weight subjects or were used as a bridging oxygenation therapy before definitive airway is secured. The major benefits of using THRIVE in obese subjects undergoing laryngeal surgery include a tubeless and uninterrupted surgical field. This advantage is especially crucial in obese patients as they tend to have limited oropharyngeal space, rendering a shared airway technically challenging for surgeons. However, concerns of potential difficult airway and shorter safe apnoeic time in the obese population limit its use. In this case, we report its use as the sole oxygenation strategy in a morbidly obese patient undergoing airway surgery. Our experience suggests that THRIVE can provide a conducive operating field and adequate oxygenation in short apnoeic laryngeal procedures in the obese population, without causing excessive hypercarbia.

## 1. Introduction

THRIVE is a novel noninvasive oxygenation technique increasingly used in the perioperative setting [1]. One major strength is its potential to delay the onset of desaturation by effectively oxygenating patients during the apnoeic period. This is achieved via the provision of continuous positive airway pressure and facilitating gaseous exchange by flow-dependent dead space flushing [2]. It has been reported to prevent desaturation for up to 65 minutes after the onset of apnoea [3], compared to six to ten minutes by conventional preoxygenation methods [4]. We describe the use of THRIVE as the mainstay oxygenation strategy in a morbidly obese patient undergoing endoscopic laryngeal microsurgery following panendoscopy.

## 2. Case Presentation

A 50-year-old gentleman presented for elective panendoscopy and biopsy of left vocal cord lesion under general anaesthesia. His background history was significant for hypertension, morbid obesity (BMI 40, weight 118kg), and possible obstructive sleep apnoea (STOPBANG 4). Airway assessment revealed a short, thick neck with the circumference of 45cm, mouth opening of two finger breaths, and Mallampati IV, indicative of possible airway difficulty.

After discussion with the surgical team, decision was made to employ a tubeless oxygenation technique using THRIVE in order to facilitate surgery in a crowded airway. The oropharynx was topicalized with 6ml of 4% lignocaine delivered via an atomiser. The patient was preoxygenated with 100% oxygen for 15 mins on the operating table, in 20-degree reverse Trendelenburg position with Optiflow™, a commercial transnasal humidified oxygen delivery system (Fisher and Paykel Healthcare Limited, Panmure, Auckland, New Zealand). Oxygen flow was gradually increased from 20L/min to 60L/min over the 15-minute preoxygenation period.

**Figure 1:**
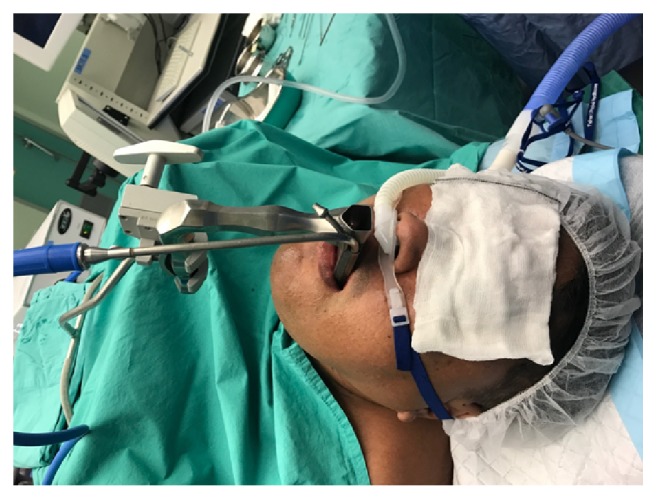
Setup of THRIVE intraoperatively, with the surgical suspension laryngoscope in place.

General anaesthesia was induced using TCI Propofol (3-4.5mcg/ml, effect site control), intravenous remifentanil infusion (0.03-0.15mcg/kg/min), and rocuronium 0.3mg/kg and maintained on total intravenous anaesthesia. Throughout induction, airway patency was achieved by maintaining a slight head tilt position, with the patient's head rested on a soft jelly ring. Oxygenation was sustained solely via THRIVE during and after induction. Once the patient was under general anaesthesia (GA), the airway was handed over to the surgeons for instrumentation.  Figure 1 shows the intraoperative setup of THRIVE. The entire surgical procedure, comprised of panendoscopy, rigid bronchoscopy, and biopsy of left vocal cord lesion, lasted 14 minutes. SpO_2_ readings were maintained above 98% throughout with oxygen flow rates of 60L/min, FiO_2_ 1.0. An arterial blood gas taken 10 minutes into the onset of apnoea showed PaCO_2_ of 65mmHg and PaO_2_ of 183mmHg. The patient was hemodynamically stable throughout surgery, with systolic blood pressures ranging between 120 and 170mmHg and heart rate between 60 and 90/min. Upon completion of the surgery, the suspension laryngoscope was removed by the surgeons. Propofol infusion was stopped and sugammadex was given as the reversal agent. Throughout the emergence process, which lasted a total of 10 mins, the patient was maintained on similar intraoperative settings of THRIVE. Prior to transfer, THRIVE was switched to a Hudson facemask delivering 8L/min of oxygen. For the next two hours, he was monitored in the recovery unit with the continued use of high-flow nasal insufflation at 20L/min, FiO_2_ 0.3, and subsequently discharged to high-dependency unit on room air. Verbal consent was obtained from the patient for publication of this case report.

## 3. Discussion

Traditionally, high-flow nasal oxygen therapy (up to 15L/min) has been achieved via regular nasal prongs. However, the main limitations of this technique are inability to humidify and heat gases, carbon dioxide accumulation resulting in excessive hypercapnia, oxygen atelectasis, patient discomfort, and mucosal desiccation. This has led to the development of devices which incorporated a heated inspiratory circuit and humidifier, allowing for higher maximal gas flows (up to 70L/min) to be delivered comfortably. Currently, THRIVE is used in settings such as the emergency room and intensive care units to treat acute respiratory failure [5, 6]. Its utility has also been extended to the perioperative context, either as a temporizing measure in challenging cases before a definitive airway is attained [7] or as a weaning strategy in patients after extubation [8].

Conventionally, the anaesthetic airway management for endoscopic microlaryngeal surgery can be divided into tube or tubeless techniques. Tube techniques involve the insertion of a microlaryngeal tube, with surgical field interference being its major drawback. Tubeless techniques include intermittent apnoeic ventilation, supraglottic or transtracheal jet ventilation via a catheter, and THRIVE. Some benefits of using THRIVE in this setting include its noninvasive nature, ability to provide an uninterrupted surgical field, prolonged safe apnoea window, and some capacity to clear CO_2_. In morbidly obese patients, a tubeless field is especially crucial as this subset of patients tend to have crowded oropharyngeal space, rendering a shared surgical field technically difficult for surgeons.

THRIVE was found to be adequate in maintaining oxygenation (up to 30mins) and preventing excessive hypercapnia (mean PaCO_2_ of 76.5mmHg) in a small Swedish series studying patients who underwent simple endoscopic laryngeal surgery [9]. While our anaesthetic technique is largely similar to that described in this study, one important difference worth mentioning is that our patient had a BMI of 40, whereas the Swedish study excluded patients with BMI >30. Obese patients are known to have a higher basal metabolic rate, poorer chest wall compliance, and lower functional residual capacity, the latter factors leading to a less efficacious preoxygenation process [10]. Collectively, these result in a shorter time to desaturation compared to individuals in the normal weight range. The fact that no desaturation occurred after 15 mins in our case suggests feasibility in applying such a strategy to morbidly obese individuals. Patel and Nouraei also reported no desaturation in their series of 25 patients which included morbidly obese patients [3]. However, although the subjects in their study included morbidly obese individuals (BMI 18- 52), THRIVE was administered as a bridging therapy until a definitive airway was attained [6]. In these studies, relative hypercapnia (up to PaCO_2_ 115mmHg) did not result in cardiac arrhythmias, excessive sympathetic stimulation, or drowsiness in the perioperative period [6, 9]. Other advantages of THRIVE include its ease of administration and low risk of barotrauma compared to conventional jet ventilation.

However, the use of THRIVE as an oxygenation strategy in airway surgery is not without limitations.

As THRIVE does not provide a secure airway, it is important that a rescue plan is devised in the event that PaO_2_ or PaCO_2_ approaches unsafe levels. We feel that infraglottic jet insufflation via a cannula cricothyroidotomy is a suitable backup plan in this clinical scenario. Ideally, the patient's airway should be thoroughly evaluated for front of neck access prior to induction. The position for cannula cricothyroidotomy can be accurately identified and marked out on the skin by performing a neck ultrasound to delineate the anatomy. Patients should be appropriately counselled for the possibility and risks of cannula cricothyroidotomy and jet ventilation. The operating theatre must also be prepped for the necessary equipment and skilled anaesthetic assistance. Other alternative rescue oxygenation techniques such as supraglottic jet ventilation, orotracheal intubation (using adjuncts such as videolaryngoscopy), or low-skill fibreoptic intubation via a supraglottic device can also be considered. However, the latter techniques are likely to disrupt the surgery and crowd the surgical field.

THRIVE has some capacity to clear CO_2_, and its rate of CO_2_ rise when used in apnoeic patients has been shown to be approximately one-third of that of classical apnoeic ventilation [3]. In the Swedish study, where THRIVE was applied to apnoeic, normal BMI patients undergoing laryngeal surgery, their mean apnoea time was 22.5 (+/-4.5) minutes, and the range of maximum PaCO_2_ attained amongst the subjects was between 44.3mmHg and 91.5mmHg [9]. Regardless, hypercapnia is a recognised limitation of THRIVE and a valid concern especially when applied to obese patients. To enhance its safety profile, one can consider regular arterial blood gas sampling intraoperatively to monitor PaCO_2_ levels and allow for timely implementation of appropriate interventions. More recently, maintenance of spontaneous respiration in laryngeal surgeries using THRIVE has been demonstrated to be a feasible technique modification, allowing for better CO_2_ control [11]. In addition, careful patient selection, maintenance of normothermia, and optimal head positioning to open up the oropharyngeal passage and facilitate air flows are all strategies that can be employed to stall CO_2_ accumulation. Patients suffering from conditions in which hypercapnia could be detrimental (e.g., pulmonary hypertension, obstructive airway diseases, and raised intracranial pressures) are not suited for THRIVE in this context.

Aspiration risks due to an unsecured airway and the potential for airway fire should also be considered. The use of prokinetics and acid neutralisers, limiting inspired oxygen concentration during laser use, and meticulous surgical techniques to minimize tissue debris are some important steps to mitigate aspiration risks. In the event of a clinically significant aspiration, the airway should be promptly secured. Video laryngoscopy, rigid or flexible fibreoptic intubation, and low-skill fibreoptic intubation are all feasible techniques to secure the airway in a patient with features of difficult airway such as in our case.

## 4. Conclusion

Our experience suggests that, with appropriate safety measures, the use of THRIVE as the primary oxygenation strategy for uncomplicated endoscopic laryngeal surgery can be safely extended to morbidly obese patients. The conduct of further studies involving obese subjects undergoing laryngeal surgery will enhance our understanding of the suitability and safety of THRIVE as a sole oxygenation tool in this select group of patients.
